# Investigation of Chinese Wolfberry (*Lycium* spp.) Germplasm by Restriction Site-Associated DNA Sequencing (RAD-seq)

**DOI:** 10.1007/s10528-018-9861-x

**Published:** 2018-06-06

**Authors:** Defang Zhang, Tao Xia, Shaofei Dang, Guanghui Fan, Zhanlin Wang

**Affiliations:** 1 Qinghai Academy of Agriculture and Forestry, Qinghai University, Xining, 810016 China; 2 State Key Laboratory of Plateau Ecology and Agriculture, Qinghai University, Xining, 810016 China; 3 Qinghai General Health Biotechnology Co., LTD, Xining, 810003 China; 4 Laboratory of Cell Biology, Research Institute of Forestry, Chinese Academy of Forestry, Xiangshan Road, Beijing, 100091 China

**Keywords:** Wolfberry, Goji, RAD-seq, Genetic background

## Abstract

Chinese wolfberry (*Lycium* spp.) is an important edible and medicinal plant, with a long cultivation history. The genetic relationships among wild *Lycium* species and landraces have been unclear for a number of reasons, which has hindered the breeding of modern Chinese wolfberry cultivars. In this study, we collected 19 accessions of Chinese wolfberry germplasm, and constructed the genetic relationship based on RAD-seq markers. We obtained 30.32 Gb of clean data, with the average value of each sample being 1.596 Gb. The average mapping rate was 85.7%, and the average coverage depth was 6.76 X. The phylogeny results distinguished all accessions clearly. All the studied landraces shared their most recent common ancestor with *L. barbarum*, which indicated that *L. barbarum* may be involved in cultivation of these landraces. The relationship of some landraces, namely the ‘Ningqi’ series, ‘Qingqi-1’ and ‘Mengqi-1,’ has been supported by the phylogeny results, while the triploid wolfberry was shown to be based on a hybrid between ‘Ningqi-1’ and a tetraploid wolfberry. This study uncovered the genetic background of Chinese wolfberry, and developed the foundation for species classification, accession identification and protection, and the production of hybrid cultivars of wolfberry.

**Electronic supplementary material:**

The online version of this article (10.1007/s10528-018-9861-x) contains supplementary material, which is available to authorized users.

## Introduction

The wolfberry (Goji) genus *Lycium* belongs to the Solanaceae family, and comprises about 80 species, which are mainly distributed in South America (30 species), southwestern North America (21 species), South Africa (17 species), and temperate Europe and Asia (Hitchcock 1932; Fukuda et al. 2001; Miller 2002). In China, there are seven species and three taxonomic varieties, mostly distributed in Gansu, Qinghai Provinces, Xinjiang, and Ningxia Autonomous regions (Kuang and Lu. 1978).

The Chinese wolfberry has been widely used as traditional medicine and healthcare products for centuries. The classic Chinese herbal texts, “Shen Nong’s Herbal Classic” and “Ben Cao Gang Mu,” have recorded its medicinal value to liver, kidney, eyes, and other organs. The latest phytochemical analyses have demonstrated that there are many valuable components in wolfberry fruits and leaves, including zeaxanthin (Inbaraj et al. 2008) and β-carotene (Peng et al. 2005), polysaccharides (Gao et al. 2008), and low molecular-weight chemicals such as betaine, cerebroside, β-sitosterol, *p*-coumaric acid, and various vitamins (Dong et al. 2009; Duan et al. 2010; Inbaraj et al. 2010).

The cultivation of Chinese wolfberry can be traced back to 1400 years ago, where selection for the edible young leaves and leaf buds was the main breeding direction, and the main propagation route was by seed. By 600 years ago, the main cultivation areas for Chinese wolfberry were in the northwest region of China, especially in Zhongning County of the Ningxia Autonomous region, where propagation was mainly based on cuttings from the excellent plants with high-quality fruits. During the long history of improvement of Chinese wolfberry, by natural selection and artificial selection, more than ten traditional landraces have been developed in the Ningxia Autonomous region, with names such as ‘Damaye,’ ‘Xiaomaye,’ ‘Black leaf,’ ‘White stick,’ and ‘Leaf medlar.’ In the past 30 years, some earlier landraces have been further improved, to exhibit superior economic characters, such as hundred grain weight, fruit size, and total sugar. Improved landraces, such as ‘Ningqi-1,’ ‘Ningqi-2,’ and ‘Ningqi-4,’ were bred from the traditional landrace ‘Damaye’ by selection from natural superior mutants, and were quickly distributed to other cultivation regions by cutting propagation. Other improved landraces include ‘Mengqi-1’ (Wang et al. 2007), developed by selection, and ‘Ningcaiqi-1,’ which was a product of hybridization and selection for superior leaf traits, while triploid wolfberry was bred by hybridizing ‘Ningqi-1’ with a tetraploid. Modern breeding methods (induced mutation breeding, space mutation breeding, and plant biotechnology) have also contributed to the development of wolfberry breeding.

However, some factors limited the modern breeding of Chinese wolfberry. Firstly, the long artificial breeding and hybridization history has had a negative influence on the conservation of superior lines, and has led to the rapid deterioration of germplasm resources (Shao et al. 2015). Secondly, there was a shortage of germplasm resources; although more than 10 landraces have been identified, only four landraces were widely planted in northwestern China, namely ‘Ningqi-1,’ ‘Ningqi-4,’ ‘Mengqi-1,’ and ‘Ningcaiqi-1’ (An et al. 2009), with the other landraces being cultivated only in restricted regions. Furthermore, most landraces were concentrated in the Ningxia Autonomous region (Hu et al. 2016). The limited genetic background of Chinese wolfberry could increase the risk of disease problems, while the relative lack of germplasm resources will not only restrict the development of wolfberry breeding, but will also slow the development of lines exhibiting high yield and high quality as demanded by consumers. Finally, the identity of individual landraces is in question, because of the absence of scientific verification. Some landraces with similar morphology have been identified as the same landrace, or the same varieties cultivated in different regions were assumed to be different landraces (An et al. 2009).

The restriction site-associated DNA (RAD-seq) method can identify polymorphic variants in genomic regions (Miller et al. 2007; Zhou et al. 2015), and has been proven to be particularly suitable for species without reference genome (Wang et al. 2017; Verdu et al. 2016). It can provide thousands of genome-scale SNP sites (Pavinato et al. 2017; Wang et al. 2017) that have successfully revealed valuable information for phylogeny (Razkin et al. 2016; Wagner and Wagner. 2017), phylogeography (Jeffries et al. 2016), population genetics (Ellegren 2014; Shih et al. 2018) and species identification (Herrera and Shank. 2016). Also, the genome-wide SNPs have provided a fundamental data resource for investigating the genetic germplasm in tea (Hua et al. 2016), grapevines (Marrano et al. 2017), peanut (Hong et al. 2015), cassava (Wosula et al. 2017), and loquat (Wang et al. 2017).

In order to detect the genetic divergence of germplasm between wolfberry landraces and their wild relatives, the third-generation sequencing technology was used, by developing RAD-seq markers, in order to investigate the genetic background of the Chinese wolfberry, and to lay down a strong foundation for the development of comprehensive Chinese wolfberry breeding project. Based on the high resolution of the RAD-seq, combined with the breeding background of Chinese wolfberry, we speculated that *L. barbarum* was the main participant during the breeding process in the studied landraces. And some of the classic cultivated landraces, like Ningqi-1 -2, -3, were involved in breeding of the other studied landraces.

## Materials and Methods

A total of 19 accessions were used in this study (Table 1), including four wild species (*L. ruthenicum* Murr., *L. barbarum* L., *L. chinense* Mill. var. *potaninii* (Pojark.) A. M. Lu, *L. yunnanense* Kuang) and a mutant with white fruits found in wild; these four species are genetically close, cross-compatible with cultivated landraces and, thus, could contribute to the improvement of Chinese wolfberry. In addition, fourteen landraces (‘Ningqi-1’ to ‘-8,’ ‘Ningcaiqi-1,’ ‘Qingqi-1,’ ‘Ningqi-v3,’ ‘Mengqi-1,’ ‘Zhongkelvchuan-1,’ and a cultivated triploid wolfberry; Table 1) have all been registered officially and are commercially planted in China. All the accessions were conserved with cutting propagation in the Germplasm Nursery of Qinghai Gouqi (Nuomuhong Co., Qinghai province), from which mature plant material was supplied.

**Table 1:** *Lycium* accessions used in this research

No.	Accession name	Origin
1	‘Ningqi-1’	Landrace ‘Damaye’
2	‘Ningqi-2’	Landrace ‘Damaye’
3	‘Ningqi-3’	The elite clone selected from *L. barbarum*
4	‘Ningqi-4’	Landrace ‘Damaye’
5	‘Ningqi-5’	Landrace ‘Ningqi-1’
6	‘Ningqi-6’	The descendant from natural hybrid of *L. barbarum*
7	‘Ningqi-7’	The elite clone selected from *L. barbarum*
8	‘Ningqi-8’	The elite clone selected from *L. barbarum*
9	‘Ningqi-v3’	–
10	‘Mengqi-1’	Plantation in Inner Mongolia
11	‘Ningcaiqi-1’	Hybrid between wild and cultivated wolfberry
12	‘Qingqi-1’	Landrace from Ningqi-1 seeds treated with mutagenesis
13	‘Zhongkelvchuan-1’	Landrace from the descendant of Ningqi-1
14	cultivated triploid wolfberry	Hybrid between Ningqi-1 and tetraploid wolfberry
15	Wild white fruit wolfberry	
16	*L. yunnanense* Kuang	
17	*L. barbarum* L.	
18	*L. chinense* Mill. var. *potaninii*	
19	*L. ruthenicum* Murr.	

Fresh leaves were collected in August 2014. All tissue samples were stored at −80 °C prior to genomic DNA isolation. Genomic DNA was extracted with the plant genomic DNA extraction kit DP305 (Tiangen, Beijing, China). DNA purity and integrity were monitored by agarose gel electrophoresis and the use of a Nanodrop spectrophotometer (NanoDrop Technologies, Wilmington, DE, USA), the latter mainly focusing on the OD_260_/OD_280_ ratio.

Based on the method of Baird et al. (2008), genomic DNA was digested with EcoRI (G∣AATTC) restriction enzyme, and the fragments were ligated to the Solexa P1 Adapter, which is complementary to the sticky ends. Subsequently, the adapter-ligated fragments were pooled, randomly sheared, and the 300–700-bp-sized fragments were selected to ligate the second adapter (P2, a Y adapter with divergent ends). Only the fragments with both P1 and P2 adapters could be amplified successfully, with amplified fragments ranging in length from 200 to 400 bp and 400–600-bp-sized fragments were collected for library construction.

To analyze the quality of the libraries, the Qubit 2.0 kit (Life Technologies, Grand Island, NY, USA) was used. Agilent 2100 (Agilent Technologies, Palo Alto, CA, USA) was used to check the insert size of the libraries after dilution of the library to 1 ng DNA/μl. Q-PCR was performed to detect the effective concentration of libraries (the effective concentration of library > 2 nM) after the fragments were appropriately inserted.

The libraries were sequenced by Illumina HiSeq 2500 platform (San Diego, CA, USA) and 125-bp paired-end reads were generated. The raw sequencing reads were firstly processed through In-House scripts to remove the bad reads that included adapter-or ploy-N containing reads, or low-quality or repetitive reads. The reads with RAD sites below a default threshold values (sequencing error rate > 1%, the depth of cover < 4 X) were also removed, because these reads could lack sufficient coverage for calling SNPs or lead to the formation of sequence error-created artifacts.

The paired-end reads of *L. barbarum* from each RAD site passing the above tests were sent to the Velvet Optimiser assembler (Zerbino and Birney. 2008). According to the record, most of the studied landraces are genetically close with *L. barbarum* (Table 1), which means that it had the largest genetic identity with the landraces, and could provide the highest sequencing contribution for sequence assemble. So the contigs of *L. barbarum* assembled by Velvet were selected as the reference genome for the next analysis.

Sequencing reads were aligned to the reference genome using BWA software (settings: mem -t 4 -k 32 –M) (Li and Durbin, 2009). Then the aligned reads were converted to BAM files using SAMtools software (Li et al. 2009). Variant allele calling was performed for each individual using SAMtools (settings: mpileup –q 1 –C 50 –S –D –m 2 –F 0.002), and SNP (Single Nucleotide Polymorphism) sites were initially filtered by vcfutils (settings: -Q 20 -d 2 -D 1000). In addition, the SNP sites with a max missing range higher than 10% were filtered out.

Based on the SNP sites, a neighbor-joining tree was generated by TreeBeST (http://treesoft.sourceforge.net/treebest.shtml). Genetic structure was inferred using the program frappe7c (http://med.stanford.edu/tanglab/software/frappe.html), which can implement an expectation maximization algorithm. To explore the convergence of individuals, the number of genetic clusters (*K*) was set from 2 to 8. The maximum iteration of the expectation–maximization algorithm was set to 100 in the frappe analysis. We computed the delta K statistics with the program admixture (http://www.genetics.ucla.edu/software/admixture/index.html) which can calculate rapidly using a fast numerical optimization algorithm.

## Results

Using the RAD-tag sequencing approach, a total of 31.26 Gb raw paired-end reads were generated from the 19 accessions. After strict filtering, 30.32 Gb clean data were retained, with the average value of raw reads being 1.65 Gb, and that of clean reads being 1.596 Gb. The average effective rate was 97.0%, the average Q20 was 93.5%, and the average Q30 was 88.2% in the 19 accessions. All information on the sequencing data is shown in Supplementary File 1.

After removing all repetitive reads, single mismatch derivatives and reads with RAD sites below the threshold value and the high-quality RAD-tags were retained. The mean number of clean reads was 6,383,952.9, and *L. barbarum* had the highest number of reads (23,122,684 reads). The mean number of duplicated reads removed was 5,667,781.9, while the mean clean duplication rate was 12.59%. The mean number of digestion reads was 5,491,317.1, and the mean digestion ratio was 96.80% (Supplementary File 2).

The paired-end reads of *L. barbarum* were assembled by Velvet 1.2.10 as the reference genome for the next analysis. The number of contigs was 880,315, which were obtained from 160,163,757 bp reads, with an average length of 295 bp.

The mapping rates of accessions reflected the similarity of accession and reference genomes, with the coverage depth demonstrating the homology with the reference genome. Statistics of the mapping rate and coverage are shown in Supplementary File 3. The average mapping rate was 85.7%, and the average coverage depth was 6.76 X. At last, the number of SNP sites that reconstructed phylogeny and genetic subdivision is 721,813. The raw and clean sequencing data were deposited in Genbank (PRJNA351229).

**Fig. 1.**
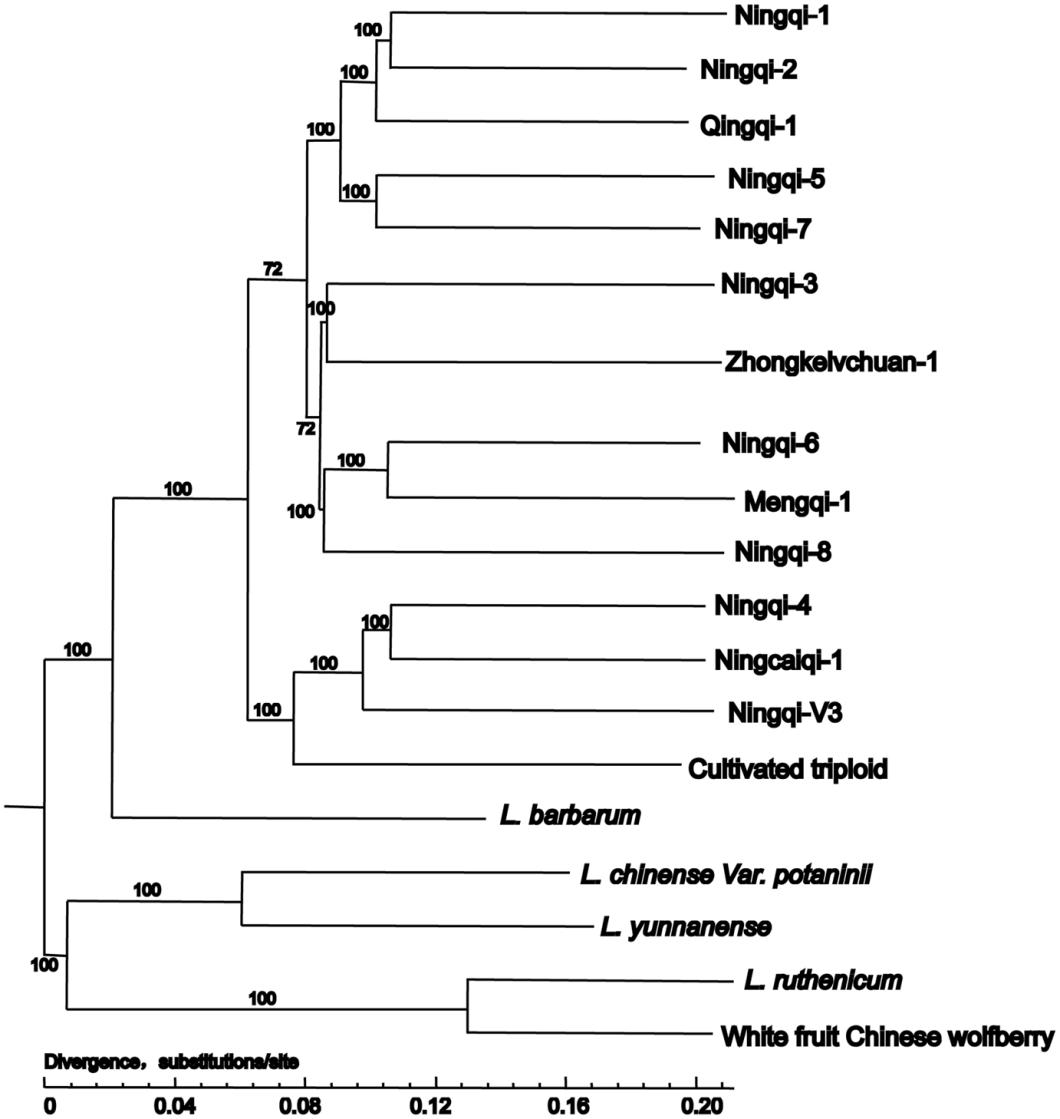
The phylogeny tree of the 19 samples based on the RAD data

The phylogeny tree was explored to determine the genetic relationships among the 19 accessions. Most accessions were separated from each other with high support (Bootstrap value = 100%). The 19 accessions were separated into two main branches: the first branch contained two clusters, one being *L. chinense* var *potaninii* and *L. yunnanense*, and the other being *L. ruthenicum* and the white fruit Chinese wolfberry. The other branch contained all the studied landraces, with *L. barbarum* located in the root of the phylogeny tree. The landraces formed three branches: cultivated triploid Chinese wolfberry clustered together with ‘Ningqi-4,’ ‘Ningcaiqi-1,’ and ‘Ningqi-3’; ‘Ningqi-1’ clustered with ‘Ningqi-2’ and ‘Qingqi-1’; and the third branch included ‘Zhongkelvchuan-1,’ ‘Mengqi-1,’ and ‘Ningqi-6’ (Fig. 1).

**Fig. 2:**
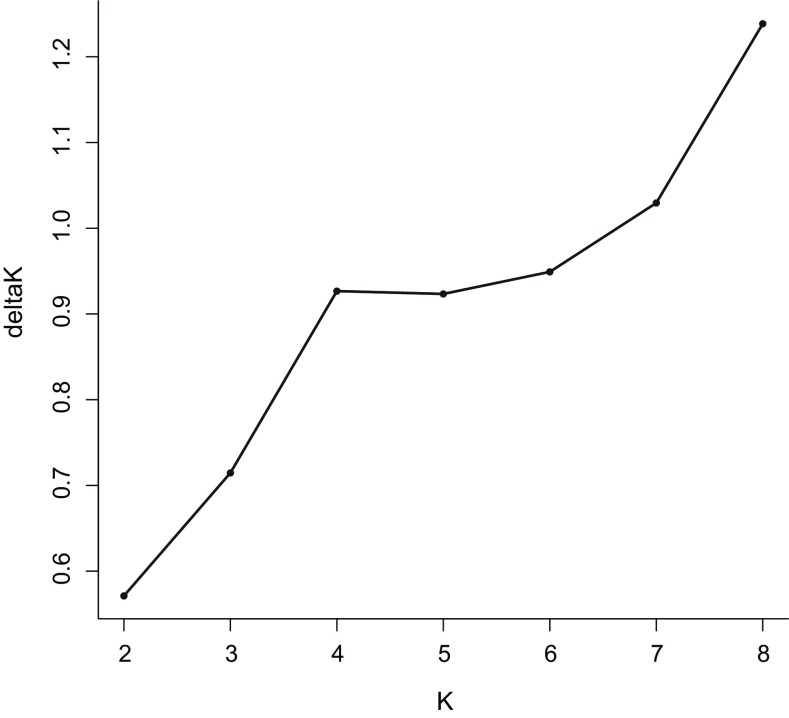
The plots for detecting the number of K groups that best fit the data

**Fig. 3:**
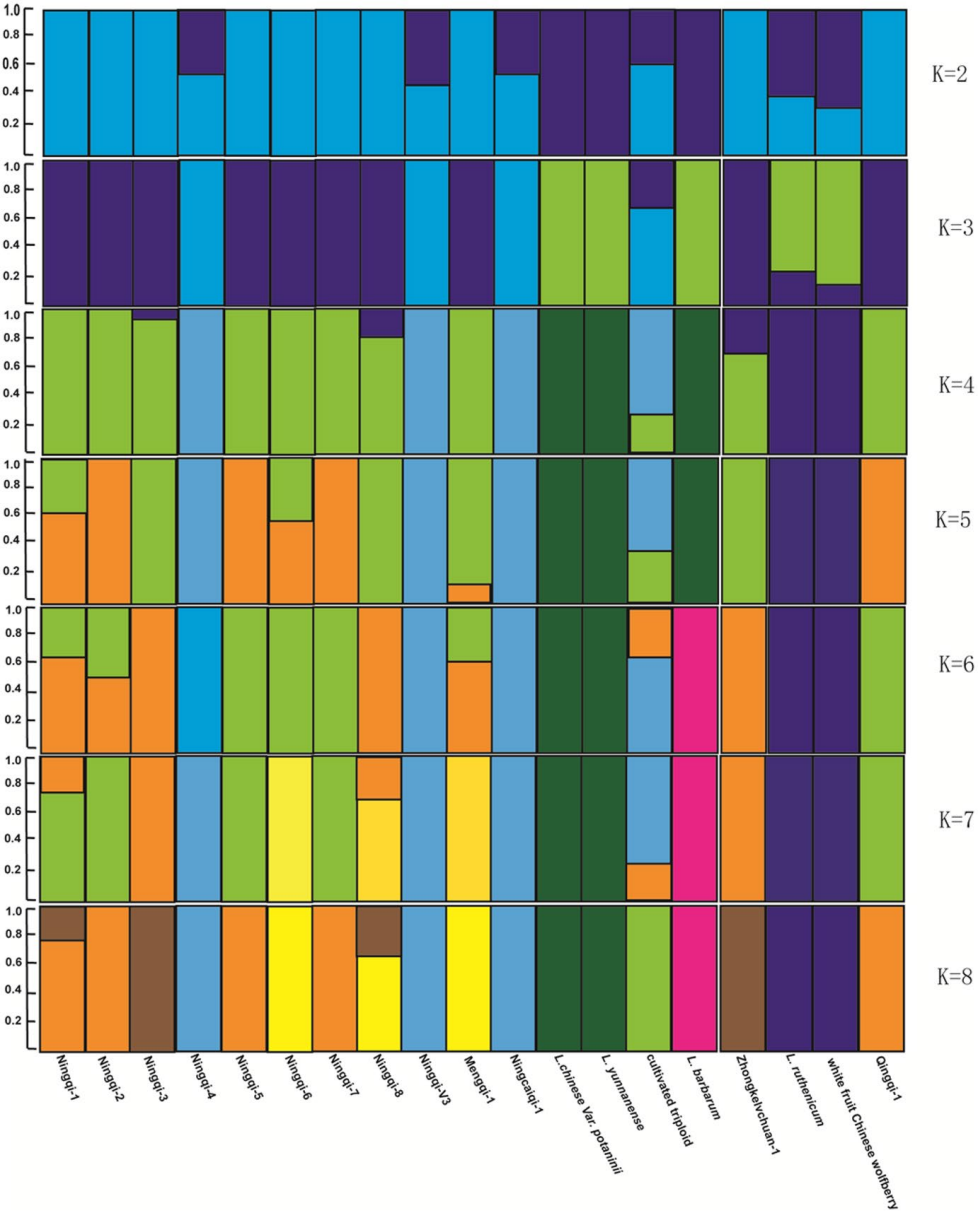
Plots of 19 individuals for K = 2 to 8, with colors representing proportion of genetic component

The genetic subdivision relationships of the landraces were uncovered by genetic structure analysis. The delta K value pointed to K = 4 (number of genetic clusters) as the strongest structure level in the data set (Fig. 2), the landraces Ningqi-1, -2, -5, -6, -7, Mengqi-1, and Qingqi-1 shared the same genetic background, while Ningqi-4, Ningqi-v3 and Ningcaiqi-1 shared the same genetic background. The same genetic subdivision was shared by *L chinense* var *potaninii* and *L. yunnanense*, and by *L. ruthenicum* and white fruit Chinese wolfberry when *K* was 4, 5, 6, 7, or 8. When *K* = 8, close relationships among the landraces were proved, such as ‘Ningqi-1,’ ‘Ningqi-2,’ and ‘Qingqi-1’; ‘Ningqi-5’ and ‘Ningqi-7’; ‘Ningqi-3’ and ‘Zhongkelvchuan-1’; ‘Ningqi-6’ and ‘Mengqi-1’; ‘Ningqi-4,’ ‘Ningqi-v3,’ and ‘Ningcaiqi-1.’ The cultivated triploid Chinese wolfberry contained two genetic components: one was from ‘Ningqi-4,’ ‘Ningqi-1,’ or ‘Ningqi-v3,’ while the other came from ‘Ningqi-3,’ which indicated the hybrid origin of triploid Chinese wolfberry (Fig. 3).

## Discussion

RAD-seq is not limited by the reference genome, and has the advantage of simple operation, with the related sequences of specific enzyme-cutting sites being scanned quickly, and rapid identification of high-density SNP-based map. In this study, the 19 accessions were selected to include the landraces that were grown over the planting area and four wild species, which may be involved in the domestication of Chinese wolfberry. Based on RAD-seq markers, the average coverage of the accession genome reached 6.76 X. The phylogeny tree obtained greater resolution (Bootstrap ≥ 72%) than had previous research, which was based on RAPD and ITS markers (Sang et al. 2010; Li et al. 2014).

A close relationship was found between *L. ruthenicum* and the white fruit Chinese wolfberry. As only a few plants were found in wild, we speculated that the white fruit Chinese wolfberry may be a mutant of *L. ruthenicum*. *L. barbarum* located in the root of clade which includes all landraces, indicating that all the studied landraces shared their most recent common ancestor with *L. barbarum*. The main distribution range of *L. barbarum* covers Ningxia, Gansu, and Qinghai area. The Ningxia area was the first cultivated region of Chinese wolfberry, so it is possible that *L. barbarum* has involved in the cultivation of some original landraces, before some landraces were cultivated in other areas. According to the literature (Li et al. 2004), the classic landraces, ‘Damaye’ and ‘Xiaomaye,’ were selected from wild Chinese wolfberry in Ningxia area.

These results also provided insights into the parentage of certain landraces. Landraces ‘Ningqi-1,’ ‘-2,’ and ‘-4’ were bred from elite lines of the classic landrace ‘Damaye,’ which, in turn, was selected from *L. barbarum*, based on the optimization of economic character. While ‘Ningqi-1’ and ‘Ningqi-2’ had a closer genetic relationship (Li et al. 2011), Ningqi-4 appeared to be quite genetically distant from the other two landraces. Some landraces were bred from *L. barbarum* directly, such as ‘Ningqi-3’ (Hu and Zhou 2005), ‘Ningqi-6,’ ‘-7,’ and ‘-8’ (Nan et al. 2014), but the results of genetic structure analysis and phylogeny did not support their close genetic relationship. This may be caused by the different parentage of landraces. Based on our field investigation, self-pollination always took place in some plants of *L. barbarum*, which could result in increased genetic variation within populations. And the wide and different environments also could increase the genetic differences among populations *L. barbarum*. Therefore, the landraces bred from different plants of *L. barbarum* could exist larger genetic differentiation.

Some landraces were developed from *L. barbarum* indirectly; ‘Ningcaiqi-1,’ for example, is a hybrid between wild and cultivated wolfberry (Li et al. 2004). ‘Ningqi-1’ was involved in the formation of some landraces, such as ‘Ningqi-5,’ ‘Qingqi-1’ (an induced mutant selected from mutagenized ‘Ningqi-1’ seeds), and ‘Zhongkelvchuan-1.’ ‘Mengqi-1’ developed from the dominant strain of the ‘Ningqi’ series (Wang et al. 2007), and our results showed that ‘Mengqi-1’ shared the same genetic background as ‘Ningqi-6,’ indicating that ‘Mengqi-1’ was bred from ‘Ningqi-6.’ A previous study had demonstrated that the triploid wolfberry was bred as a hybrid of ‘Ningqi-1’ and tetraploid wolfberry (An et al. 1998). Our results found that the triploid wolfberry possessed a genetic subdivision of ‘Ningqi-1’ or ‘Ningqi-3,’ and one of ‘Ningqi-4,’ ‘Ningcaiqi-1,’ or ‘Ningqi-v3.’

All the landraces had the common ancestor with *L. barbarum*, which could have been the cause of the close genetic relationship between the landraces. The wild wolfberry had greater genetic diversity than did the landraces. The low diversity of the landraces may be a consequence of long-term artificial directional selection, eliminating the lines (and genes) unsuitable for crop production and resulting in similar morphology (“convergent evolution”) among landraces from different parentages. From the perspective of long-term development of Chinese wolfberry, in order to prevent quality deterioration caused by inbreeding, the Chinese wolfberry industry should introduce more wolfberry germplasm material into the breeding program to enrich the Chinese wolfberry gene pool.

## Electronic supplementary material

Below is the link to the electronic supplementary material.

Supplementary material 1 (DOCX 16 kb)

Supplementary material 2 (DOCX 16 kb)

Supplementary material 3 (DOCX 16 kb)
