# Supplementary material for: Investigation of Chinese Wolfberry (Lycium spp.) Germplasm by Restriction Site-Associated DNA Sequencing (RAD-seq)
Source: Biochem Genet. 2018 Jun 6;56(6):575–85. doi: 10.1007/s10528-018-9861-x (PMC6223726; doi:10.1007/s10528-018-9861-x)
Supplement: Supplementary file 1 — Supplementary material 1 (DOCX 16 kb) [file 10528_2018_9861_MOESM1_ESM.docx]

Additional file 1. Sequencing data and quality of all samples.

| Sample | Raw Base(bp) | Clean  Base(bp) | Effective Rate(%) | Error Rate(%) | Q20(%) | Q30(%) | GC  Content(%) |
| --- | --- | --- | --- | --- | --- | --- | --- |
| Ningqi-1 | 1,499,998,000 | 1,475,505,000 | 98.37 | 0.04 | 94.04 | 89.11 | 36.59 |
| Ningqi-2 | 1,499,997,250 | 1,478,301,750 | 98.55 | 0.04 | 93.17 | 87.52 | 36.98 |
| Ningqi-3 | 1,280,514,500 | 1,264,067,500 | 98.72 | 0.04 | 93.86 | 88.77 | 36.96 |
| Ningqi-4 | 1,670,943,750 | 1,651,045,750 | 98.81 | 0.04 | 93.64 | 88.41 | 37.64 |
| Ningqi-5 | 1,499,997,750 | 1,483,349,500 | 98.89 | 0.04 | 93 | 87.1 | 37.42 |
| Ningqi-6 | 1,499,998,750 | 1,481,332,500 | 98.76 | 0.04 | 93.28 | 87.62 | 37.2 |
| Ningqi-7 | 1,500,000,500 | 1,479,985,250 | 98.67 | 0.04 | 93.17 | 87.46 | 37.47 |
| Ningqi-8 | 1,142,151,000 | 1,126,288,500 | 98.61 | 0.04 | 93.69 | 88.47 | 37.97 |
| Ningqi-v3 | 1,499,998,500 | 1,475,830,250 | 98.39 | 0.04 | 93.86 | 88.77 | 37.12 |
| Mengqi-1 | 1,010,137,250 | 995,071,500 | 98.51 | 0.04 | 93.7 | 88.52 | 37.14 |
| Ningcaiqi-1 | 1,858,824,750 | 1,821,934,250 | 98.02 | 0.04 | 93.74 | 88.47 | 37.73 |
| *L. chinense*. var. *potaninii* | 1,499,999,500 | 1,474,541,250 | 98.3 | 0.04 | 93.91 | 88.79 | 38.56 |
| *L. yunnanense* | 1,644,821,150 | 1,139,491,436 | 69.28 | 0.04 | 93.69 | 88.28 | 38.29 |
| cultivated triploid Chinese wolfberry | 1,305,726,750 | 1,287,966,250 | 98.64 | 0.04 | 93.66 | 88.38 | 37.67 |
| *L. barbarum* | 5,868,777,750 | 5,780,671,000 | 98.5 | 0.04 | 92.87 | 86.9 | 37.97 |
| Zhongkelvchuan -1 | 1,200,473,750 | 1,183,782,250 | 98.61 | 0.04 | 93.55 | 88.27 | 37.59 |
| *L. ruthenicum* | 1,040,554,000 | 1,025,283,750 | 98.53 | 0.04 | 93.7 | 88.47 | 38.62 |
| Wild white fruit Chinese wolfberry | 1,237,208,750 | 1,215,657,250 | 98.26 | 0.04 | 93.65 | 88.39 | 38.04 |
| Qingqi-1 | 1,500,200,250 | 1,483,671,500 | 98.9 | 0.04 | 93.25 | 87.62 | 37.22 |
| Mean value | 1,645,280,205.3 | 1,595,988,233.5 | 97.0 | 0.04 | 93.5 | 88.2 | 37.6 |
