# Supplementary material for: Investigation of Chinese Wolfberry (Lycium spp.) Germplasm by Restriction Site-Associated DNA Sequencing (RAD-seq)
Source: Biochem Genet. 2018 Jun 6;56(6):575–85. doi: 10.1007/s10528-018-9861-x (PMC6223726; doi:10.1007/s10528-018-9861-x)
Supplement: Supplementary file 2 — Supplementary material 2 (DOCX 16 kb) [file 10528_2018_9861_MOESM2_ESM.docx]

Table 2. RAD tags of all samples.

| Sample | Clean reads | Removed duplication reads | Clean duplication rate (%) | Digestion reads | Digestionratio (%) |
| --- | --- | --- | --- | --- | --- |
| Ningqi-1 | 5,902,020 | 4,964,957 | 15.88 | 4,757,196 | 95.82 |
| Ningqi-2 | 5,913,207 | 5,424,222 | 8.27 | 5,283,518 | 97.41 |
| Ningqi-3 | 5,056,270 | 4,154,219 | 17.84 | 4,025,130 | 96.89 |
| Ningqi-4 | 6,604,183 | 5,689,358 | 13.85 | 5,517,064 | 96.97 |
| Ningqi-5 | 5,933,398 | 5,437,931 | 8.35 | 5,345,335 | 98.30 |
| Ningqi-6 | 5,925,330 | 5,641,230 | 4.79 | 5,544,641 | 98.29 |
| Ningqi-7 | 5,919,941 | 5,427,806 | 8.31 | 5,308,737 | 97.81 |
| Ningqi-8 | 4,505,154 | 3,641,143 | 19.18 | 3,486,828 | 95.76 |
| Ningqi-v3 | 5,903,321 | 5,406,646 | 8.41 | 5,220,290 | 96.55 |
| Mengqi-1 | 3,980,286 | 3,484,231 | 12.46 | 3,363,697 | 96.54 |
| Ningcaiqi-1 | 7,287,737 | 6,101,868 | 16.27 | 5,921,105 | 97.04 |
| *L. chinense* var. *potaninii* | 5,898,165 | 5,031,473 | 14.69 | 4,830,049 | 96.00 |
| *L. yunnanense* | 4,557,966 | 4,045,648 | 11.24 | 3,927,801 | 97.09 |
| cultivated triploid Chinese wolfberry | 5,151,865 | 4,266,468 | 17.19 | 4,116,943 | 96.50 |
| *L. barbarum* | 23,122,684 | 22,032,652 | 4.71 | 21,317,936 | 96.76 |
| Zhongkelvchuan -1 | 4,735,129 | 3,828,488 | 19.15 | 3,677,767 | 96.06 |
| *L. ruthenicum* | 4,101,135 | 3,368,497 | 17.86 | 3,238,824 | 96.15 |
| Wild white fruit Chinese wolfberry | 4,862,629 | 4,070,927 | 16.28 | 3,872,502 | 95.13 |
| Qingqi-1 | 5,934,686 | 5,670,093 | 4.46 | 5,579,662 | 98.41 |
| Mean value | 6,383,952.9 | 5,667,781.9 | 12.59 | 5,491,317.1 | 96.80 |
