# Supplementary material for: Investigation of Chinese Wolfberry (Lycium spp.) Germplasm by Restriction Site-Associated DNA Sequencing (RAD-seq)
Source: Biochem Genet. 2018 Jun 6;56(6):575–85. doi: 10.1007/s10528-018-9861-x (PMC6223726; doi:10.1007/s10528-018-9861-x)
Supplement: Supplementary file 3 — Supplementary material 3 (DOCX 16 kb) [file 10528_2018_9861_MOESM3_ESM.docx]

Table 3.The mapping rate and sequencing depth.

| Sample | Total reads | Mapped reads | Mapping rate(%) | coverage depth(X) |
| --- | --- | --- | --- | --- |
| Ningqi-1 | 11,804,040 | 10,168,542 | 86.14 | 7.09 |
| Ningqi-2 | 11,826,414 | 9,919,327 | 83.87 | 7.21 |
| Ningqi-3 | 10,112,540 | 8,522,794 | 84.28 | 6.08 |
| Ningqi-4 | 13,208,366 | 11,297,824 | 85.54 | 6.1 |
| Ningqi-5 | 11,866,796 | 10,122,945 | 85.3 | 7.24 |
| Ningqi-6 | 11,850,660 | 10,087,038 | 85.12 | 7.13 |
| Ningqi-7 | 11,839,882 | 10,109,022 | 85.38 | 7.26 |
| Ningqi-8 | 9,010,308 | 7,657,467 | 84.99 | 5.82 |
| Ningqi-v3 | 11,806,642 | 10,002,215 | 84.72 | 5.65 |
| Mengqi-1 | 7,960,572 | 6,608,445 | 83.01 | 5.41 |
| Ningcaiqi-1 | 14,575,474 | 12,657,246 | 86.84 | 6.72 |
| Qingqi-1 | 11,869,372 | 10,237,047 | 86.25 | 7.23 |
| Triploid Chinese wolfberry | 10,303,730 | 8,832,189 | 85.72 | 5.24 |
| *L. chinense* var. *potaninii* | 11,796,330 | 10,811,612 | 91.65 | 6.65 |
| *L. yunnanense* | 9,115,932 | 8,309,015 | 91.15 | 6.02 |
| *L. barbarum* | 46,245,368 | 41,912,578 | 90.63 | 14.65 |
| Zhongkelvchuan -1 | 9,470,258 | 7,997,231 | 84.45 | 5.78 |
| *L. ruthenicum* | 8,202,270 | 6,723,146 | 81.97 | 5.53 |
| Wild white fruit Chinese wolfberry | 9,725,258 | 7,908,812 | 81.32 | 5.63 |
| Average value | 12,767,905.9 | 11,046,552.4 | 85.7 | 6.76 |
